# Supplementary material for: End Sequence Analysis Toolkit (ESAT) expands the extractable information from single-cell RNA-seq data
Source: Genome Res. 2016 Oct;26(10):1397–410. doi: 10.1101/gr.207902.116 (PMC5052061; doi:10.1101/gr.207902.116)
Supplement: Supplemental Material [file supp_gr.207902.116_Supp_Materials.tar.gz › ESAT-esat_v0.1/doc/html/enumbroad_1_1pda_1_1seq_1_1graph_1_1_chromosome_with_bubbles_j_graph_t_1_1_edge_source_type.html]

ESAT: broad.pda.seq.graph.ChromosomeWithBubblesJGraphT.EdgeSourceType Enum Reference


ESAT


- Main Page
- Related Pages
- Packages
- Classes
- Files

- Class List
- Class Index
- Class Hierarchy
- Class Members

All Classes Namespaces Files Functions Variables Enumerator Pages

- broad
- pda
- seq
- graph
- ChromosomeWithBubblesJGraphT
- EdgeSourceType

Public Attributes |
List of all members

broad.pda.seq.graph.ChromosomeWithBubblesJGraphT.EdgeSourceType Enum Reference

|  |  |
| --- | --- |
| Public Attributes | |
|  | SPLICED |
|  | |
|  | PAIRED |
|  | |
|  | PAIRED\_SUPPORT |
|  | |

## Member Data Documentation

broad.pda.seq.graph.ChromosomeWithBubblesJGraphT.EdgeSourceType.PAIRED

broad.pda.seq.graph.ChromosomeWithBubblesJGraphT.EdgeSourceType.PAIRED\_SUPPORT

broad.pda.seq.graph.ChromosomeWithBubblesJGraphT.EdgeSourceType.SPLICED

---

The documentation for this enum was generated from the following file:

- java/broad/pda/seq/graph/ChromosomeWithBubblesJGraphT.java


---

Generated on Fri Aug 15 2014 15:11:02 for ESAT by  

 1.8.7
